# Revised computational metagenomic processing uncovers hidden and biologically meaningful functional variation in the human microbiome

**DOI:** 10.1186/s40168-017-0231-4

**Published:** 2017-02-08

**Authors:** Ohad Manor, Elhanan Borenstein

**Affiliations:** 1 Department of Genome Sciences, University of Washington, Seattle, WA 98195 USA; 2 Department of Computer Science and Engineering, University of Washington, Seattle, WA 98195 USA; 3 Santa Fe Institute, Santa Fe, NM 87501 USA

**Keywords:** Functional metagenomics, Gut microbiome, Functional variation

## Abstract

**Background:**

Recent metagenomic analyses of the human gut microbiome identified striking variability in its taxonomic composition across individuals. Notably, however, these studies often reported marked functional uniformity, with relatively little variation in the microbiome’s gene composition or in its overall metabolic capacity.

**Results:**

Here, we address this surprising discrepancy between taxonomic and functional variations and set out to track its origins. Specifically, we demonstrate that the functional uniformity observed in microbiome studies can be attributed, at least partly, to common computational metagenomic processing procedures that mask true functional variation across microbiome samples. We identify several such procedures, including commonly used practices for gene abundance normalization, mapping of gene families to functional pathways, and gene family aggregation. We show that accounting for these factors and using revised metagenomic processing procedures uncovers such hidden functional variation, significantly increasing observed variation in the abundance of functional elements across samples. Importantly, we find that this uncovered variation is biologically meaningful and that it is associated with both host identity and health.

**Conclusions:**

Accurate characterization of functional variation in the microbiome is essential for comparative metagenomic analyses in health and disease. Our finding that metagenomic processing procedures mask underlying and biologically meaningful functional variation therefore highlights an important challenge such studies may face. Alternative schemes for metagenomic processing that uncover this hidden functional variation can facilitate improved metagenomic analysis and help pinpoint disease- and host-associated shifts in the microbiome’s functional capacity.

**Electronic supplementary material:**

The online version of this article (doi:10.1186/s40168-017-0231-4) contains supplementary material, which is available to authorized users.

## Background

The human gut microbiota—the assemblage of bacterial species that inhabit our intestinal tract—performs a variety of crucial metabolic processes, interacts with our immune system, and protects us from opportunistic pathogens [1]. Comparisons of these microbial communities across individuals have identified marked variation in their taxonomic composition at various phylogenetic levels [2, 3]. In contrast, however, examining the set of genes encoded in the metagenome revealed that the functional capacity of these communities is fairly similar across individuals, especially when viewed at the pathway level [4–7]. For example, analysis of gut microbiome samples in the Human Microbiome Project (HMP) demonstrated that broad metabolic categories, such as central carbohydrate metabolism, cofactor and vitamin biosynthesis, and ATP synthesis, are similarly abundant across all individuals [6]. Similarly, analysis of gut samples from 1135 Dutch individuals demonstrated that the abundances of gene ontology molecular function categories were stable across all individuals [7], and an analysis of the microbiomes of obese and lean twins showed relatively constant abundances of clusters of orthologous functions despite variable taxonomic profiles [4]. In agreement with these findings, a study that compared the accuracy of using either taxonomic or functional profiles to classify microbiome samples to the specific body site or host of origin indeed concluded that using functional profiles does not improve classification accuracy [8]. This functional uniformity may reflect some constraints on community-level functional capacity required for survival in the gut environment [9, 10]. Nevertheless, numerous recent studies have found that variation in the functional capacity of the microbiome can impact human health [11, 12]. Evidently, even though the functional capacity of the gut microbiome is generally similar across individuals, subtle variation in its ability to produce or degrade certain metabolites could have a marked effect on the well-being of the host. For example, studies found that increased production of the short-chain fatty acid butyrate by the microbiota is associated with reduced inflammation, potentially promoting gut health [13, 14]. Other studies found that variation in the amount of microbiota-produced trimethylamine is associated with the risk of cardiovascular disease [15, 16]. Detection and accurate characterization of the functional variation in the human microbiome is therefore essential for future studies and could facilitate pinpointing specific functional capacities associated with host health and developing microbiome-based precision therapies.

Importantly, recent analyses of metagenomic pipelines have demonstrated that using different computational processing schemes can markedly impact the obtained functional profiles. For example, a recent study comparing different approaches of mapping sequencing reads to functional categories concluded that the variation introduced by the choice of mapping approach outweighed underlying variation between sampled individuals [17]. In addition, we recently found that a routinely used metagenomic normalization method could introduce spurious variation into the functional profile of microbiome samples [18]. It is therefore plausible that commonly employed computational methods for processing metagenomic data could similarly impact the functional variation observed in the microbiome.

Here, we set out to examine this hypothesis and to detect specific computational practices used in the processing of metagenomic data that could mask important variation across microbiome functional profiles. We specifically identify four different such practices, namely relative normalization, the inclusion of prevalent bacterial genes, gene aggregation, and gene mapping schemes, that contribute to functional variation masking. We further present a revised metagenomic processing procedure, based on both novel and previously introduced approaches, and show that it can uncover a more variable, health-related, and host-specific functional profile. These findings suggest that the common perception of a uniform functional capacity in the microbiome can be attributed, at least partly, to commonly employed computational procedures. Moreover, given the extensive ongoing efforts to identify disease-associated functional imbalances in the microbiome, highlighting procedures that hinder such efforts by potentially masking true biologically relevant variation is a timely and crucial step towards an improved understanding of the role of the microbiome’s functional capacity in human health.

## Results

### Normalization of functional abundance profiles

Metagenomic analyses that aim to characterize the functional capacity of the microbiome often normalize the number of reads that map to each gene family by the total number of mapped reads in the sample, representing the amount of each gene family as its *relative* abundance in the sample. This *relative normalization* scheme allows researchers to compare the abundance profiles of different functions across samples with varying sequencing depths [6, 10]. Importantly, however, as noted above, this normalization scheme introduces *spurious* functional variation in gene family abundances across metagenomic samples. Specifically, we have recently shown that several sample-specific factors, including the average size of genomes in a sample and the phylogenetic distance of these genomes to available reference genomes, bias relative normalization-based profiles [18]. Here, we therefore wished to examine whether these biases not only result in spurious variation but also distort and mask true underlying variation.

**Fig. 1:**
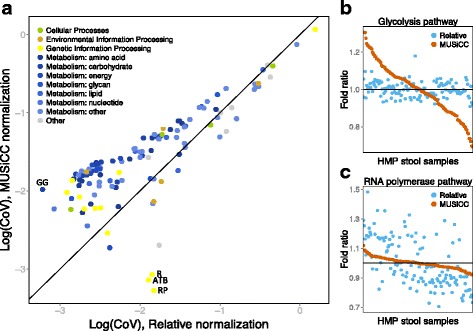
Relative normalization masks functional variability. **a** Shown is a scatter plot of the coefficient of variation (CoV) in the abundance of pathways across gut samples from the Human Microbiome Project, normalized either by relative normalization (*x-axis*) or by MUSiCC-based normalization (*y-axis*). Each point represents a single pathway. *GG* glycolysis/gluconeogenesis, *R* ribosome, *ATB* aminoacyl-tRNA biosynthesis; *RP* RNA polymerase. **b**, **c** Scatter plots showing the fold change in abundance of each sample from the average abundance across all gut samples, normalized either by relative normalization (*blue*) or by MUSiCC-based normalization (*orange*), for the glycolysis/gluconeogenesis (**b**) and the RNA polymerase (**c**) pathways. Each *point* represents a single sample and samples are ordered according to their MUSiCC-based fold change

To this end, we used MUSiCC (*M*etagenomic *U*niversal *Si*ngle-*C*opy *C*orrection), a marker gene-based method we developed previously to correct relative normalization-induced biases [18] (and see also Refs. [19, 20]), and compared the functional variation observed across samples when applying MUSiCC to the variation observed when using standard relative normalization. MUSiCC uses a set of universal single-copy marker genes as a yardstick to calibrate gene family abundances across samples, representing the prevalence of each gene family in a sample as the average copy number of this gene family across all genomes in the sample rather than as its relative abundance. MUSiCC-based normalization therefore corrects the biases induced by relative normalization and may restore and reveal the true underlying variation. Indeed, applying these two normalization schemes to 134 gut samples from the HMP [6], we found that MUSiCC significantly increased the average functional variation observed in the abundances of Kyoto Encyclopedia of Genes and Genomes (KEGG) pathways across samples (*P* < 10^−12^, paired Student’s *t* test; Fig. 1a and see also Additional file 1: Figure S1, Additional file 2: Figure S2, Additional file 3: Tables S1–S2; see “Methods”). Interestingly, the pathways that exhibited the largest increase in variation when applying MUSiCC were mostly associated with carbohydrate, amino acid, and energy metabolism, a finding that is in line with the role of such pathways in coping with different metabolic requirements that may be induced by changes in the gut environmental conditions. For example, the *glycolysis/gluconeogenesis* pathway exhibited a 3.4-fold increase in variation across samples when applying MUSiCC (Fig. 1b). This pathway has been previously linked to Crohn’s disease [21], mirroring the observed imbalance in energy metabolism in mouse models of inflammatory bowel disease [22]. Additional examples include the *tyrosine metabolism* pathway (2.7-fold increase; linked to drug efficacy [23]), the *pentose phosphate* pathway (2.8-fold increase; linked to obesity [24]), and the *propanoate metabolism* pathway (2.5-fold increase; linked to IBD [21]). Conversely, the 3 pathways that had the most notable decrease in variation when applying MUSiCC were all genetic information processing pathways such as the *RNA polymerase* pathway (Fig. 1c), the *tRNA biosynthesis* pathway, and the *ribosome* pathway, which represent common bacterial processes that are less likely to vary across samples.

To further confirm that MUSiCC reveals true underlying variation, we additionally conducted a large-scale simulation analysis, examining observed variation in simulated communities of varying complexity (see Additional file 4: Text S1). We found that MUSiCC indeed recovered the true functional variation present across samples markedly better than relative normalization (Additional file 5: Figure S3). Moreover, below we further report that MUSiCC can improve the detection of disease-associated functional shifts and the identification of individuals based on their microbiomes’ functional profiles. Combined, these findings suggest that the variation uncovered by MUSiCC likely represents real and biologically relevant variation.

### Inclusion of prevalent bacterial gene families

Clearly, whereas some gene families are encoded by a limited number of bacterial species and represent unique metabolic capacities or adaptations, other gene families are extremely prevalent and can be found in almost any bacterial genome. Such prevalent genes (e.g., those involved in transcription and translation or in basic metabolic processes) represent core microbial functions with relatively little dependency (or impact) on the environment the species inhabit. The abundances of these functions in the microbiome are thus arguably not necessarily informative of specific gut environment or host state and are similarly less likely to vary across samples. Likewise, other gene families may not be associated with core microbial functions but rather with processes crucial for surviving specifically in the gut environment (e.g., anaerobic respiration or fermentation) and will therefore be prevalent not across all genomes but rather in gut-dwelling species. These genes will similarly vary relatively little across gut samples and will have little impact on the host. Considering the abundance of these '*all genome-prevalent'* and '*gut genome-prevalent'* genes when estimating the abundance of various functional pathways in the gut microbiome could therefore mask relevant variation in the abundance of other, more specific genes that are more likely to be associated with the host state (and whose variation across samples is therefore of most interest).

**Fig. 2:**
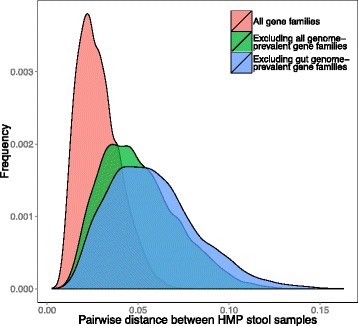
Filtering prevalent gene families increases functional variability. Shown are the distributions of pairwise Bray-Curtis distances in the pathway-level abundance profiles between different samples of the gut microbiome, including all gene families (*red*), or excluding *all genome-prevalent* (*green*), or excluding *gut genome-prevalent* (*blue*) gene families

To examine the impact of including all genome-prevalent genes in metagenomic functional profiles’ calculation, we first compiled a list of genes that were found to have a similar copy number across all 2337 bacterial genomes in KEGG (see “Methods” and Additional file 3: Table S3). We further compiled a list of gut genome-prevalent genes, including all genes that have a similar copy number across the genomes of a previously characterized set of 177 gut-dwelling bacterial species [25] (see “Methods” and Additional file 3: Tables S4 and S5). Since our metric of variation depends on the average abundance of the pathway (see “Methods”), here, we did not examine the overall variation in the abundance of each pathway across individuals (since excluding genes is expected to lower the average abundance of pathways) but rather calculated all pairwise distances *between* the pathway-level functional profiles of the various individuals. Indeed, we found that excluding all genome-prevalent gene families significantly increased the average pairwise distance between individuals (*P* < 10^−15^, Student’s *t* test; Fig. 2; see “Methods”). Notably, a Procrustes analysis demonstrated that excluding all genome-prevalent gene families increased inter-individual differences without significantly altering the structure of the distance matrix (*P* < 0.001, Procrustes permutation test; Additional file 6: Figure S4). Moreover, excluding also gut genome-prevalent gene families further increased the average pairwise distance between individuals (*P* < 10^−15^, compared both to excluding all genome-prevalent and to considering all gene families; Fig. 2). Combined, these results suggest that inter-individual differences in the metagenome are at least partly masked by the inclusion of genes that are invariable across genomes. Excluding such gene families may therefore facilitate the identification of subtle differences between individuals’ microbiomes that might play a role in host phenotypic variation.

### Aggregation of gene families into pathways

To gain a more interpretable and biologically meaningful result in comparative functional metagenomic analysis, researchers routinely aggregate gene families into higher-level functional categories such as metabolic or structural pathways. The obtained pathway-level profiles provide a broader view of the functional capacity encoded in the metagenome and can potentially reduce noise stemming from sequencing and annotation errors. However, higher-level aggregation can also mask important and biologically relevant variation at lower functional resolutions. For example, different functional modules that are annotated with the same pathway often represent alternative strategies employed by different species for performing some functional task. Accordingly, such pathway-level aggregation could completely mask changes in the strategy employed by the community (induced by shifts in the composition of the community) with potentially interesting functional consequences. Indeed, examining the correlation between the abundances of the different gene families included in the same KEGG pathway across HMP gut metagenomic samples, we found that a substantial fraction (9.5% on average) of gene family pairs were negatively correlated (*R* < −0.3; *P* < 0.0005, Pearson’s correlation test). This observation is in agreement with the hypothesis that in different microbial communities, species may employ different functional strategies that could be reflected in a more variable metagenome across gut samples.

**Fig. 3:**
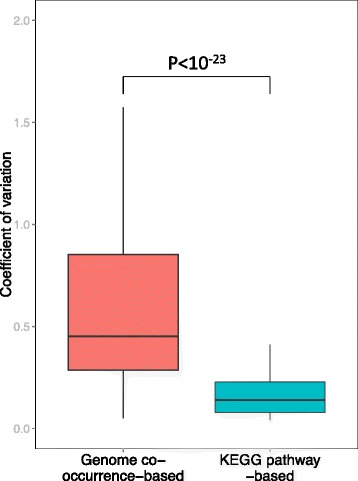
Grouping gene families into KEGG pathway-based aggregates decreases functional variability. Shown are boxplots of the distributions of coefficient of variation (CoV) values in the abundance of different gene family aggregates across gut metagenomic samples from the Human Microbiome Project. Genome co-occurrence-based aggregates (*red*) show significantly higher values of CoV compared to KEGG pathway-based aggregates (*cyan*; *P* < 10^−23^, Wilcoxon rank-sum test)

An alternative approach is therefore to define functional aggregates as groups of gene families that tend to co-occur in microbial *genomes* and that can therefore be considered as cohesive microbial genome functional units (see, for example, Ref. [26]). Using such *genome co-occurrence-based* aggregates to quantify the abundance of functional groups would likely better capture functional variation induced by shifts in community composition across a set of metagenomic samples and could improve our ability to detect interesting changes in the functional capacity of the microbiome. To examine this hypothesis, we defined a list of genome co-occurrence-based aggregates based on the similarity in the presence/absence pattern of gene families across all bacterial genomes in KEGG (see “Methods”). We found that the observed functional variation of genome co-occurrence-based aggregates across gut metagenomic samples was significantly higher than the variation observed in *KEGG pathway-based* aggregates (*P* < 10^−23^, Student’s *t* test; Fig. 3). Importantly, this finding could not be explained solely by the fact that genome co-occurrence-based aggregates tended to exhibit a different size distribution than *KEGG* pathway-based aggregates (Additional file 7: Figure S5).

### Quantification of the contribution of shared gene families to pathway abundance

As discussed above, in most functional metagenomic studies, gene abundances are converted into pathway-level profiles, representing the aggregated abundances of gene families associated with each pathway. The mapping from gene families to pathways, however, is not unique as many gene families are associated with multiple pathways. The abundance of such a *shared* gene family is usually uniformly assigned to all pathways with which it is associated, either by dividing the abundance of this gene family uniformly across these pathways (referred to below as *uniform fractional* mapping) or by adding the entire gene family abundance to each pathway (referred to below as *uniform complete* mapping; see, for example, Ref. [27]). However, while shared gene families could indeed in principle play a role in multiple pathways, in any given sample, the observed abundance of a gene may be linked more to its role in one pathway vs. another and should therefore contribute differentially to the abundance of each pathway. Moreover, as microbiomes vary in their functional capacities, the abundance of a certain shared gene family may be driven by different pathways in different metagenomic samples and the way its abundance is assigned to the various pathways with which it is associated should be therefore sample-specific.

To address this challenge, here, we introduce a novel pathway assignment scheme that takes into account the overall functional profile of the metagenome when determining the contribution of each shared gene family to the pathways with which it is associated. Specifically, the functional profile of each sample is first used to calculate the average abundance of all *non-shared* gene families associated with each pathway. Since non-shared gene families are not affected by the multiple mapping issues discussed above, they can serve as an unbiased estimate for the presence of each pathway in the sample. These calculated average abundances of non-shared gene families are then used as *support values* for each pathway, and the abundance of each shared gene family is partitioned among the pathways with which it is associated according to the ratio between the support values of these pathways (see full details in “Methods”). The abundance of each pathway is finally defined as the sum of all the partial contributions from the abundances of shared gene families calculated above and the entire abundances of all the non-shared gene families associated with this pathway. An implementation of this scheme, termed *EMPANADA* (*E*vidence-based *M*etagenomic *P*athway *A*ssignment using ge*N*e *A*bundance *DA*ta) is available online (see “Methods”).

**Fig. 4:**
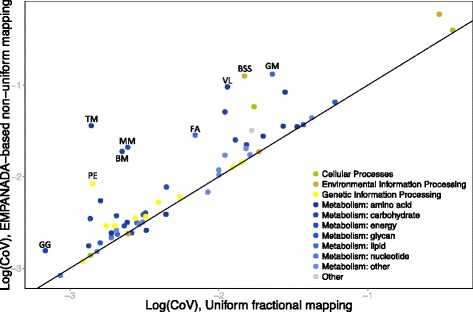
Non-uniform and sample-specific mapping of gene families to pathways increases functional variability. Shown is a scatter plot of the coefficient of variation (*CoV*) in the abundance of pathways across gut samples from the Human Microbiome Project, where gene families are mapped to pathways either by uniform fractional mapping (*x-axis*) or by EMPANADA-based non-uniform mapping (*y-axis*). Each *point* represents a single pathway. *GG* glycolysis/gluconeogenesis, *PE* protein export, *TM* tyrosine metabolism, *BM* butyrate metabolism, *MM* methane metabolism, *FA* fatty acid metabolism *VL* valine, leucine, and isoleucine degradation, *BSS* bacterial secretion system, *GM* glycerolipid metabolism

To examine whether such sample-specific, non-uniform mapping of the abundance of shared gene families impacts the observed variation in pathway-level functional profiles, we applied this scheme and the two uniform mapping schemes described above (i.e*.*, fractional and complete) to HMP gut metagenomic samples and compared the observed variation in the resulting profiles. We found that applying the EMPANADA-based non-uniform mapping scheme resulted in a marked increase in variability for several pathways (Fig. 4, and see also Additional file 8: Figure S6). Specifically, a significant increase in variation across samples was observed in the *bacterial secretion system* and *protein export* pathways, an intriguing finding considering the importance of these pathways for microbe-microbe and microbiome-host interactions and the potential variability that different gut environments may exert on such interactions [28–30]. Other metabolic pathways that exhibited a substantial increase in variation when applying non-uniform mapping were the *butyrate metabolism* and *methane metabolism* pathways, both of which represent species-specific functional capacities [31, 32] that were found to be differentially abundant in inflammation-related diseases [21].

### Accounting for masked functional variation reveals hidden disease associations

One of the main goals of characterizing the functional capacity of the microbiome is to identify functions whose prevalence in the metagenome is significantly associated with host health. We therefore next set out to examine whether the above approaches to uncover masked variation impact our ability to detect such associations and whether uncovered variation is accordingly biologically relevant. To this end, we analyzed previously obtained metagenomic samples from a large Chinese type 2 diabetes (T2D) cohort [11] using either *standard* metagenomic processing or a *revised* pipeline that implements the various schemes for uncovering hidden variation described above (see “Methods”). Specifically, the standard pipeline used relative abundance normalization, considered all gene families associated with each pathway, and applied uniform mapping of gene families to pathways, whereas the revised pipeline, in contrast, used MUSiCC for normalization, filtered prevalent gene families, and applied the non-uniform mapping scheme described above. We then quantified the association of each functional pathway with T2D observed in each pipeline.

**Fig. 5:**
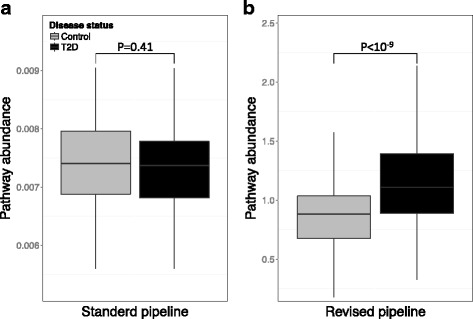
Applying a revised pipeline to uncover masked functional variation improves identification of disease-associated functional shifts. Shown are boxplots of the abundance of the cysteine and methionine metabolism pathway across type 2 diabetes (T2D) case (*black*) and control (*gray*) samples using either the standard (**a**) or revised (**b**) metagenomic bioinformatic processing pipelines

We found that the disease-association scores calculated based on functional profiles obtained by the two pipelines were overall consistent (*R* = 0.52, *P* < 10^−7^, Pearson correlation test across all functional pathways; Additional file 9: Figure S7). Notably, however, several pathways showed a markedly different disease-association pattern when the revised pipeline was applied. Specifically, the *cysteine and methionine metabolism* pathway (ko00270) showed the largest difference, exhibiting a slight (and non-significant) *depletion* in T2D samples when using the standard pipeline but a strong *enrichment* in T2D samples when using the revised pipeline (Fig. 5; *P* < 10^−10^, Wilcoxon rank-sum test). Notably, in an independent comparative study of the microbiomes of European T2D patients, cysteine and methionine metabolism was indeed one of the most significant T2D-associated pathways identified [12]. A similar trend (i.e., a significant association with T2D only when applying the revised pipeline) was also found for several other pathways such as the *phosphotransferase system (PTS*; ko02060*)* and the *Biofilm formation-vibrio cholerae* (ko05111; see Additional file 9: Figure S7), both of which were found to be associated with high levels of sugar in the blood [12]. Several other pathways showed T2D-association only when using the standard pipeline although the association of some of these pathways with T2D has been indeed challenged by other studies (see Additional file 4: Text S1, Additional file 9: Figure S7, and Additional file 3: Table S6). These results suggest that uncovering functional variation that is often masked by standard metagenomic bioinformatic processing can improve our ability to identify important associations between the functional capacity of the microbiome and disease.

### Unmasking hidden functional variation facilitates unique identification of individuals based on their functional microbiome profiles

Recently, several studies examined whether an individual’s metagenome can serve as a “fingerprint,” uniquely identifying that individual over time [33, 34]. These studies analyzed microbiome samples from a group of individuals and constructed a *metagenomic code*, aiming to identify a small subset of microbiome features that could be matched to samples from the same individual obtained at a later time point. Notably, however, this code was constructed using only *taxonomic* information, rather than *functional* information. Here, we therefore wanted to examine whether the observed variation in the functional makeup of the microbiome (especially after unmasking hidden variation as described above) is consistent across time and can similarly serve as a unique fingerprint for identifying individuals.

To this end, we analyzed 36 individuals from the HMP cohort for which gut microbiome samples from two consecutive visits were available (similar to the data used in Ref. [34]). We first evaluated whether inter-individual variation in metagenomic functional profiles is preserved between the two visits. We used the pathway-level profile of each sample and compared the pairwise distances between individuals in the first visit to the pairwise distances between individuals in the second visit. Using a Mantel test to assess the agreement between the first and second inter-individual distances, we found that these pairwise distances were significantly correlated (*R* = 0.18, Pearson’s correlation; *P* < 0.03, Mantel test). Next, we examined whether applying the schemes described above for uncovering hidden variation improves the consistency between the two visits. Indeed, we found that applying MUSiCC normalization resulted in a markedly increased correlation of *R* = 0.34 (*P* < 10^−3^; Mantel test) and that removing all genome-prevalent gene families from the MUSiCC-corrected functional profile and using genomic co-occurrence-based aggregation of gene families further increased the correlation to *R* = 0.36 and *R* = 0.41, respectively (*P* < 10^−3^ for both; Mantel test).

Given the temporal consistency in inter-individual variation, we finally examined whether metagenomic functional profiles can uniquely identify an individual in the second visit based on samples collected in the first visit. We used a similar overall approach to Franzosa et al. [34] but used functional profiles (in contrast to the taxonomic profiles used in that study) to construct *functional metagenomic codes* for all individuals based on samples from the first visit. Next, we used these functional metagenomic codes to identify individuals in the second visit by finding the first visit sample with the closest code (see “Methods”). We found that when using a functional metagenomic code that consists of only 7 gene families (similar to the number of features used in Ref. [34]), ~81% (29 of 36) of individuals could be accurately identified. Moreover, applying MUSiCC to the functional profile before constructing functional metagenomic codes increased the accuracy to 86% (31/36). Notably, such accuracy in identifying individuals is markedly higher than the reported accuracy obtained by using only 16S-based species-level information (~30%; see Ref. [34]) and is comparable to the best reported accuracy obtained using strain-specific sequence-based markers (86%; see Ref. [34]). Put differently, variation in revised functional profiles is as informative as variation in taxonomy (even when using strain-level resolution), suggesting that the perceived uniformity in functional data compared to taxonomic data is a potential misconception. Furthermore, examining functional metagenomic codes with varying numbers of gene families (ranging from 1 to 20) showed an overall improved identification accuracy with MUSiCC-based normalization (*P* < 0.001, paired Student’s *t* test; Additional file 10: Figure S8). For example, functional metagenomic codes using the MUSiCC-normalized abundances of 20 gene families reached an identification accuracy of 94% (34/36) compared to 91% (33/36) with relative normalization. These findings suggest that metagenomic functional profiles of individuals are consistent across time and that a functional metagenomic code can uniquely identify individuals at extremely high accuracy.

## Discussion

The composition of species in the gut microbiome likely reflects, at least partly, a well-defined set of ecological constraints required for surviving in the human intestinal tract [35]. Since in many cases multiple species can perform the same function, it is plausible that the functional capacity of the microbiome will remain relatively constant in the face of variation in species composition. Nevertheless, the observation of uniformity in functional microbiome profiles (especially in the face of substantial taxonomic variation) has become a canonical observation in microbiome research and has puzzled the microbiome research community. In the analyses above, we systematically analyzed the effect of several routinely used metagenomic processing procedures on observed functional variation and showed that at least some of the uniformity observed in the microbiome’s functional capacity across individuals is due to these common computational practices. This apparent uniformly hinders ongoing efforts to understand the impact of the microbiome’s functional capacity on its host and to reliably detect disease-associated shifts in this capacity. Acknowledging the role that computational processing may play in masking relevant and informative functional variation is accordingly a timely message. Importantly, we found that using a revised computational pipeline for functional metagenomic processing significantly increased the variation observed across metagenomic gut samples, and that this increased variation is consistent over time and can facilitate the identification of disease-associated pathways and the construction of a functional microbiome fingerprint.

Interestingly, the various practices we discuss include both practical and conceptual factors. For example, we showed that two common bioinformatic processing schemes, namely relative normalization of abundances and uniform assignment of the abundance of gene families to pathways, dramatically mask observed functional variation. The application of bioinformatic tools that were developed specifically with functional metagenomic processing in mind, such as MUSiCC (for normalization; [18]) or EMPANADA (for mapping), could therefore alleviate these problems. In contrast, including genome-prevalent gene families in the calculation of functional profiles or aggregating gene families based on potentially arbitrary functional annotations (rather than based on genomic co-occurrence patterns) reflect conceptual data analysis approaches that are not necessarily unjustified but do impact measured variation. Our findings therefore do not imply necessarily that alternative approaches should always be used, but instead highlight the effect such analysis approaches have on downstream functional analysis, allowing researchers to better evaluate the choices made in processing and mining metagenomic data.

## Conclusions

This study was based on the hypothesis that the compositional functional uniformity routinely observed in gut microbiome studies may be, at least partly, attributed to commonly used metagenomic computational procedures. Indeed, we were able to identify several computational practices that mask true and biologically meaningful functional variation observed across metagenomic samples, and to show that a revised metagenomic analysis pipeline that accounts for these factors can obtain a more variable, disease-associated, and host-specific microbiome functional profile. Such alternative schemes for metagenomic processing that can accurately characterize functional variation in the microbiome are crucial for comparative metagenomic analyses of the microbiome in health and disease and can facilitate pinpointing important shifts in the microbiome’s functional capacity.

## Methods

### Microbiome data

Data from the Human Microbiome Project (HMP) were downloaded from the Data Analysis and Coordination Center website (http://www.hmpdacc.org/), including KEGG [36, 37] Orthology group (KO) abundances and sample metadata. Data from the type 2 diabetes (T2D) cohort were downloaded from the human gut microbiome integrated reference catalog [25] (http://meta.genomics.cn/meta/dataTools), including KO abundances and sample metadata.

### Calculating pathway abundance profiles and functional variation

The abundance of each pathway in each sample was calculated as the sum of abundances (either relative- or MUSiCC-normalized) of all gene families (i.e*.,* KOs) associated with this pathway. For each KEGG pathway, the variation in the profile was calculated as the *coefficient of variation* (CoV), defined as the standard variation in the abundance across samples divided by the mean abundance. Functional distance between pathway-level abundance profiles of different gut samples was calculated using the Bray-Curtis distance metric.

### Defining all-genome and gut genome-prevalent genes

All genome-prevalent genes were defined as KOs with copy number CoV < 1.5 across all 2337 bacterial genomes in KEGG. Notably, while the availability of additional genomes is expected to reduce the number of all genome-prevalent genes (in this study 1168 such KOs were defined; Additional file 3: Table S3), a rarefication analysis has confirmed that the rate at which the number of genes shrinks slows down with the number of genomes used, suggesting that it may eventually plateau (see Additional file 11: Figure S9). Gut genome-prevalent genes were similarly defined across a previously published reduced set of 177 gut-dwelling bacterial genomes [25] (see Additional file 3: Table S4 and Table S5).

### Aggregating gene families based on pairwise correlation in bacterial genomes

In order to construct genome co-occurrence-based functional aggregates, the pairwise Jaccard distance between pairs of gene families (i.e., KOs) was calculated as the fraction of bacterial genomes in KEGG in which both KOs are present out of all genomes in which at least one of the KOs is present. Using this distance metric, hierarchical clustering was performed using the complete linkage method. The number of final genome co-occurrence-based aggregates was chosen to be similar to the number of KEGG pathways.

### Uniform and non-uniform mapping of gene families to pathways

In the uniform mapping schemes, all KOs associated with each pathway in the KEGG database contributed equally to its final abundance. For uniform fractional mapping, the abundance of the KO was uniformly divided among all pathways with which it is associated, while for uniform complete mapping, the abundance of the KO was added in its entirety to all pathways with which it is associated. In the non-uniform mapping scheme (implemented as EMPANADA; see below), the *support* of each pathway was defined as the average abundance of all its non-shared KOs (i.e., KOs that are associated only with this pathway in the KEGG database). Next, the abundance of each shared gene family was partitioned among the pathways with which it is associated according to the ratio between the support values of these pathways. The abundance of each pathway was finally defined as the sum of all the partial contributions from the abundances of shared gene families calculated above and the entire abundances of all the non-shared gene families associated with this pathway. This analysis was restricted to pathways that have at least 10 non-shared KOs and an overall mean relative abundance >0.05%.

### EMPANADA software implementation and distribution

The gene family to pathway mapping tool, EMPANADA, was implemented in Python and is available for download at http://elbo.gs.washington.edu/software.html. EMPANADA is also available as a pip-installable python package (i.e., pip install –U empanada) and the source code is available on GitHub (https://github.com/borenstein-lab/empanada).

### Constructing and evaluating a function-based metagenomic code

For each individual, a functional metagenomic code was constructed, by identifying the set of functional elements that best separated their first visit sample from all other first visit samples. Specifically, for each individual, the set of gene families (i.e., KOs) that had the highest relative abundance in this individual compared to all other individuals were first identified. The set of the top 20 KOs that had the largest ratio between this individual and the next highest were further selected, defining the metagenomic code of that individual as this set of 20 marker KOs. This process was repeated for all individuals, resulting in a final set of 720 KOs that were selected as markers for at least one individual. Next, *agreement scores* (ranging from 0 to 20) between the abundance profiles of samples in the second visit to the metagenomic codes generated from the first visit were computed. Specifically, for each of these 720 KOs, the individual with the highest relative abundance for this KO in the second visit was identified, and the agreement score for the individual who included this KO in their metagenomic code was increased by 1. Finally, each second visit sample was assigned to the first visit sample with which it had the highest agreement score. The identification accuracy was defined as the number of individuals whose second visit sample was correctly assigned to their first visit sample. This process was also repeated using codes of sizes 1–19 KOs, and using MUSiCC-based normalization (Additional file 10: Figure S8).

## Additional files

Additional file 1: Figure S1. Relative normalization masks functional variation of pathways in HMP gut samples. Shown is the same plot shown in Fig. 1a, where each pathway is additionally marked by a numeric identifier corresponding to its identifier in Additional file 3: Table S1.  (PDF 251 kb)
Additional file 2: Figure S2. Relative normalization masks functional variation of modules in HMP gut samples. Shown is a scatter plot similar to Fig. 1a but on the *module *level, where each module is additionally marked by a numeric identifier corresponding to its identifier in Additional file 3: Table S2.  (PDF 251 kb)
Additional file 3: Tables S1–S6. Supplementary tables for this study. (XLSX 337 kb)
Additional file 4: Text S1. Supplementary information for this study. (PDF 322 kb)
Additional file 5: Figure S3. Relative normalization impacts functional variation of pathways in simulated microbial communities. Shown is a scatter plot illustrating the CoV of each pathway calculated for 100 simulated metagenomic communities comprised of 100 reference genomes with varying abundances. The true CoV is shown on the x-axis, while the calculated CoV after relative normalization (red) or MUSiCC (blue) is shown on the y-axis. GG: Glycolysis/Gluconeogenesis; R: Ribosome. (PDF 251 kb)
Additional file 6: Figure S4. Excluding *all genome-prevalent* gene families increases inter-individual differences in the metagenome while maintaining the overall structure of inter-individual distances. Shown is a Procrustes analysis of the metagenomic functional inter-individuals differences. Two principal coordinate (PCoA) matrices were constructed (using the Bray-Curtis distance) for pathway-level profiles either including (orange circles) or excluding (blue triangles) *all genome-prevalent* gene families. Arrows connect the two profiles of the same individual. Using a permutation-based approach, the two PCoAs were found to be significantly similar (R=0.91, *P*<0.001). (PDF 251 kb)
Additional file 7: Figure S5. Metagenomic functional variation in gene family aggregates based on KEGG pathways or microbial genome co-occurrence. Shown are boxplots of the distributions of Coefficient of Variation (*CoV*) values in the abundance of *genome co-occurrence-based* gene family aggregates and *KEGG pathway-based* gene family aggregates across gut samples from HMP. Since the average size of aggregates (*i.e.*, the number of gene families assigned to each aggregate) is different between the two aggregation schemes, a permuted assignment of gene families to aggregates was performed for each scheme (*i.e.*, preserving the size of the aggregates) and the CoV was calculated for the permuted aggregates. Notably, *KEGG pathway-based* aggregates did not show significantly higher values of CoV than *KEGG pathway-based* permuted aggregates (*P*=0.38, Wilcoxon rank-sum test), while *genome co-occurrence-based* aggregates showed significantly higher values of CoV than *genome co-occurrence-based* permuted aggregates (*P*<10^-4^, Wilcoxon rank-sum test). (PDF 251 kb)
Additional file 8: Figure S6. Non-uniform and sample-specific mapping of gene families to pathways increases variability in the abundance of pathways in HMP gut samples. Shown is the same plot as in Fig. 4, with pathways additionally marked by a numeric identifier corresponding to their identifier in Additional file 3: Table S1, comparing the EMPANADA-based non-uniform mapping strategy with the *uniform fractional* (**a**) and *uniform complete* (**b**) mapping schemes.  (PDF 251 kb)
Additional file 9: Figure S7. Association of pathways to type 2 diabetes (T2D) with and without accounting for factors masking functional variability. Shown is a scatter plot of the differential abundance score (*W*-statistic of the Wilcoxon rank-sum test) of each pathway when comparing T2D cases and controls, using either the *standard *(x-axis) or *revised *(y-axis) metagenomic bioinformatic processing pipelines. Each point represents a single pathway and is additionally marked by its KEGG identifier. The dashed red lines represent the FDR<0.05 significance cutoff for each metagenomic processing pipeline. (PDF 251 kb)
Additional file 10: Figure S8. Function-based metagenomic code. Shown is a plot of the accuracy of identification of an individual’s microbiome sample based on a previously obtained sample from the same individual. The identification was done using functional metagenomic codes of varying sizes (1-20 gene families), based on the gene family abundance profiles normalized with either relative (red) or MUSiCC (cyan) normalization procedures. (PDF 251 kb)
Additional file 11: Figure S9. Rarefication analysis of the number of all-genome prevalent genes as a function of the number of genomes used. *All-genome prevalent* genes were defined as KOs with a copy number CoV < 1.5 across bacterial genomes used. (PDF 251 kb)

## Abbreviations

COV: Coefficient of variation
EMPANADA: Evidence-based Metagenomic Pathway Assignment using geNe Abundance DAta
HMP: Human microbiome project
KEGG: Kyoto Encyclopedia of Genes and Genomes
KO: KEGG orthology group
MUSiCC: Metagenomic Universal Single-Copy Correction
T2D: Type 2 diabetes
