# Supplementary figures and images for: Revised computational metagenomic processing uncovers hidden and biologically meaningful functional variation in the human microbiome
Source: Microbiome. 2017 Feb 8;5:19. doi: 10.1186/s40168-017-0231-4 (PMC5299786; doi:10.1186/s40168-017-0231-4)

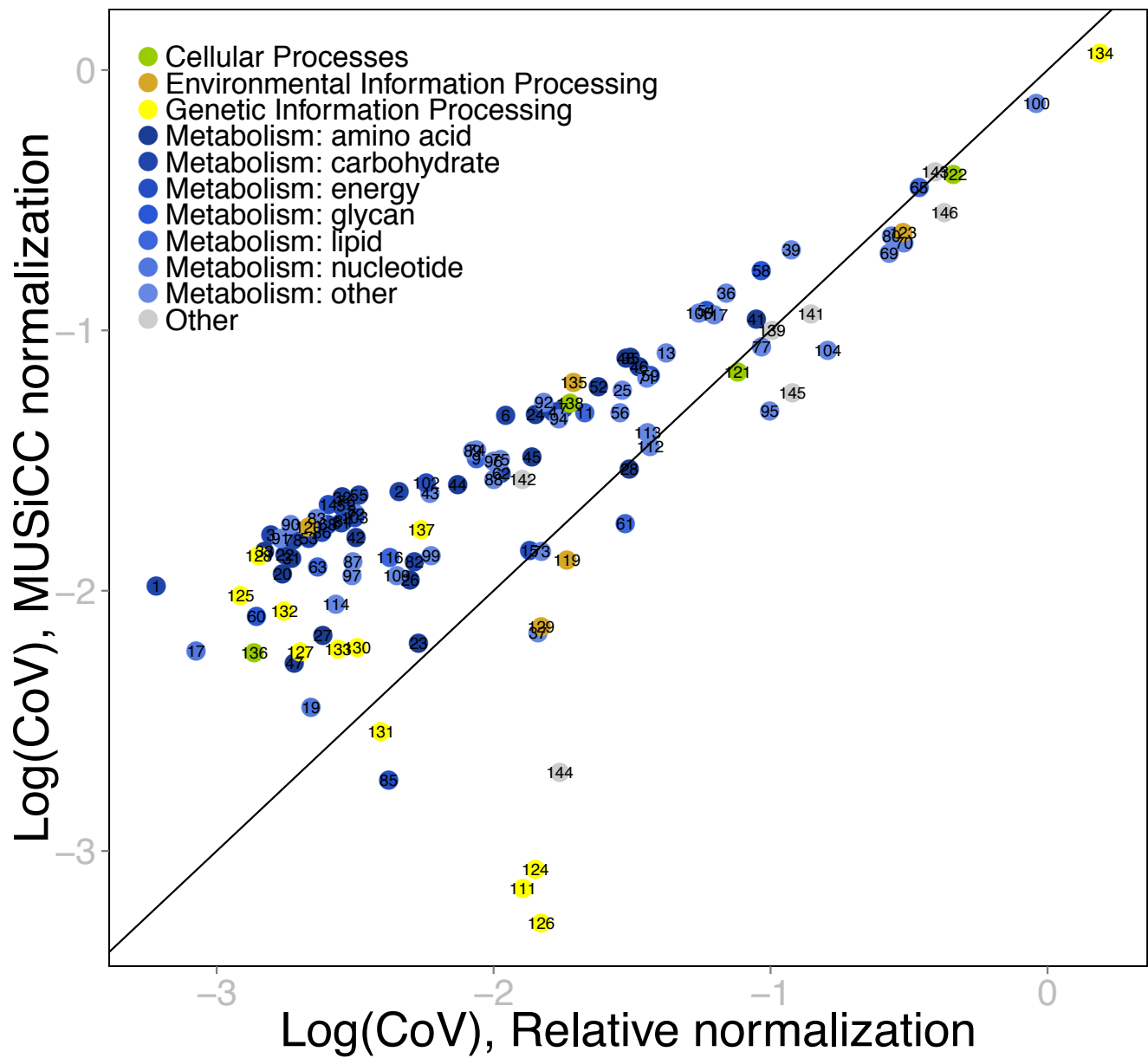

**Figure S1**

Supplement: Additional file 1: Figure S1. — Relative normalization masks functional variation of pathways in HMP gut samples. Shown is the same plot shown in Fig. 1a, where each pathway is additionally marked by a numeric identifier corresponding to its identifier in Additional file 3: Table S1. (PDF 251 kb) [file 40168_2017_231_MOESM1_ESM.pdf]

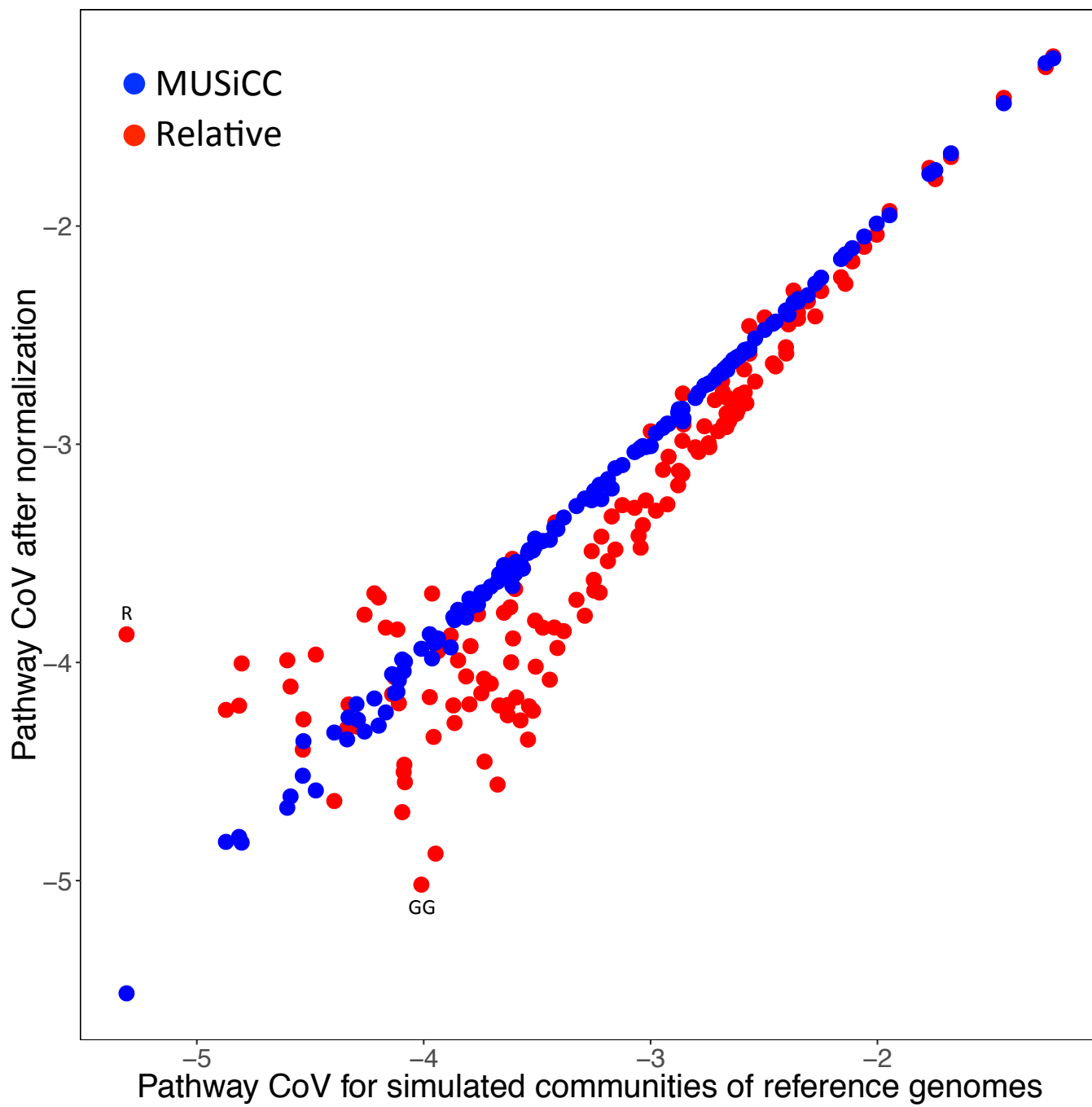

**Figure S3**

Supplement: Additional file 5: Figure S3. — Relative normalization impacts functional variation of pathways in simulated microbial communities. Shown is a scatter plot illustrating the CoV of each pathway calculated for 100 simulated metagenomic communities comprised of 100 reference genomes with varying abundances. The true CoV is shown on the x-axis, while the calculated CoV after relative normalization (red) or MUSiCC (blue) is shown on the y-axis. GG: Glycolysis/Gluconeogenesis; R: Ribosome. (PDF 251 kb) [file 40168_2017_231_MOESM5_ESM.pdf]

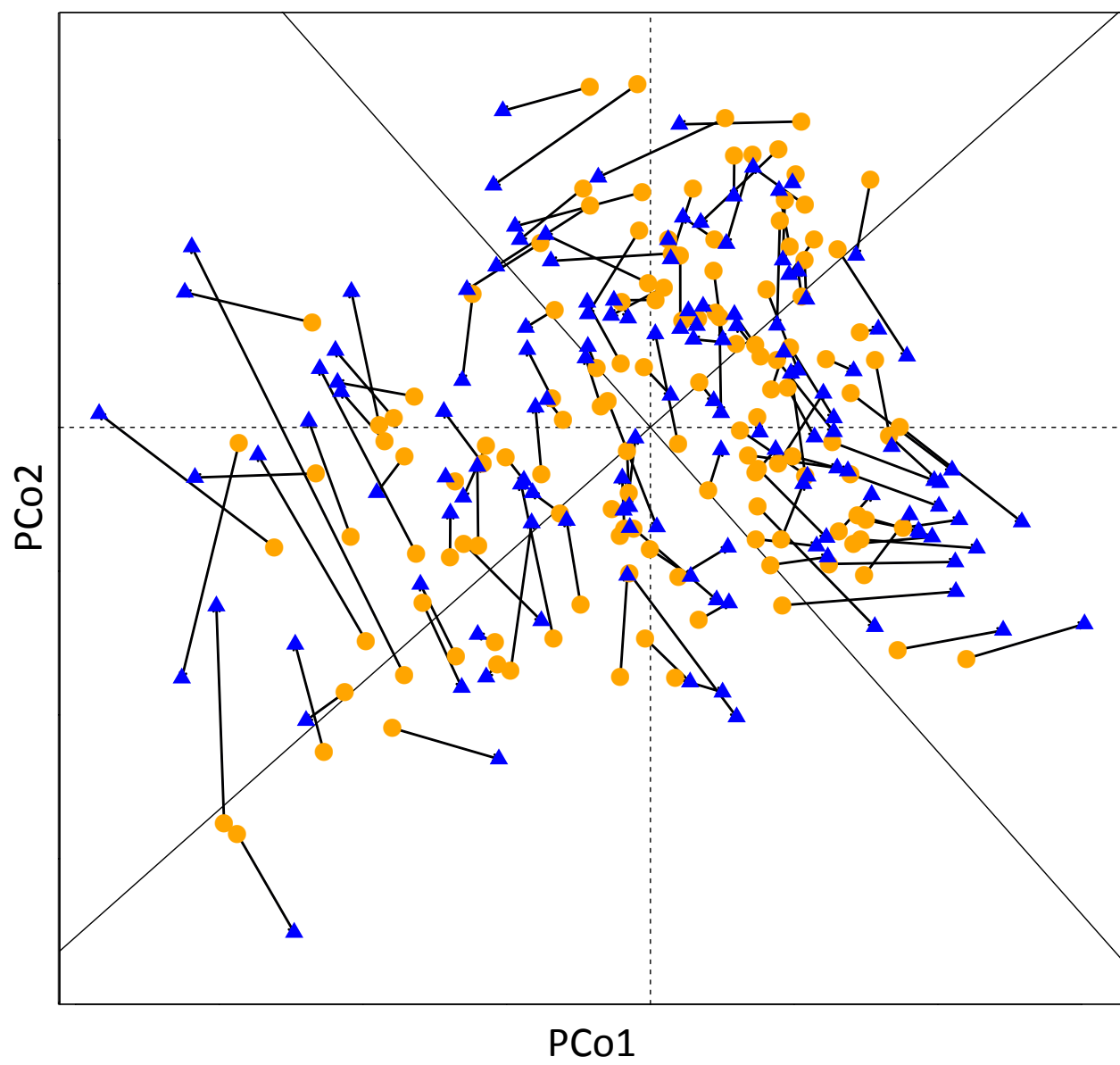

**Figure S4**

Supplement: Additional file 6: Figure S4. — Excluding all genome-prevalent gene families increases inter-individual differences in the metagenome while maintaining the overall structure of inter-individual distances. Shown is a Procrustes analysis of the metagenomic functional inter-individuals differences. Two principal coordinate (PCoA) matrices were constructed (using the Bray-Curtis distance) for pathway-level profiles either including (orange circles) or excluding (blue triangles) all genome-prevalent gene families. Arrows connect the two profiles of the same individual. Using a permutation-based approach, the two PCoAs were found to be significantly similar (R=0.91, P<0.001). (PDF 251 kb) [file 40168_2017_231_MOESM6_ESM.pdf]

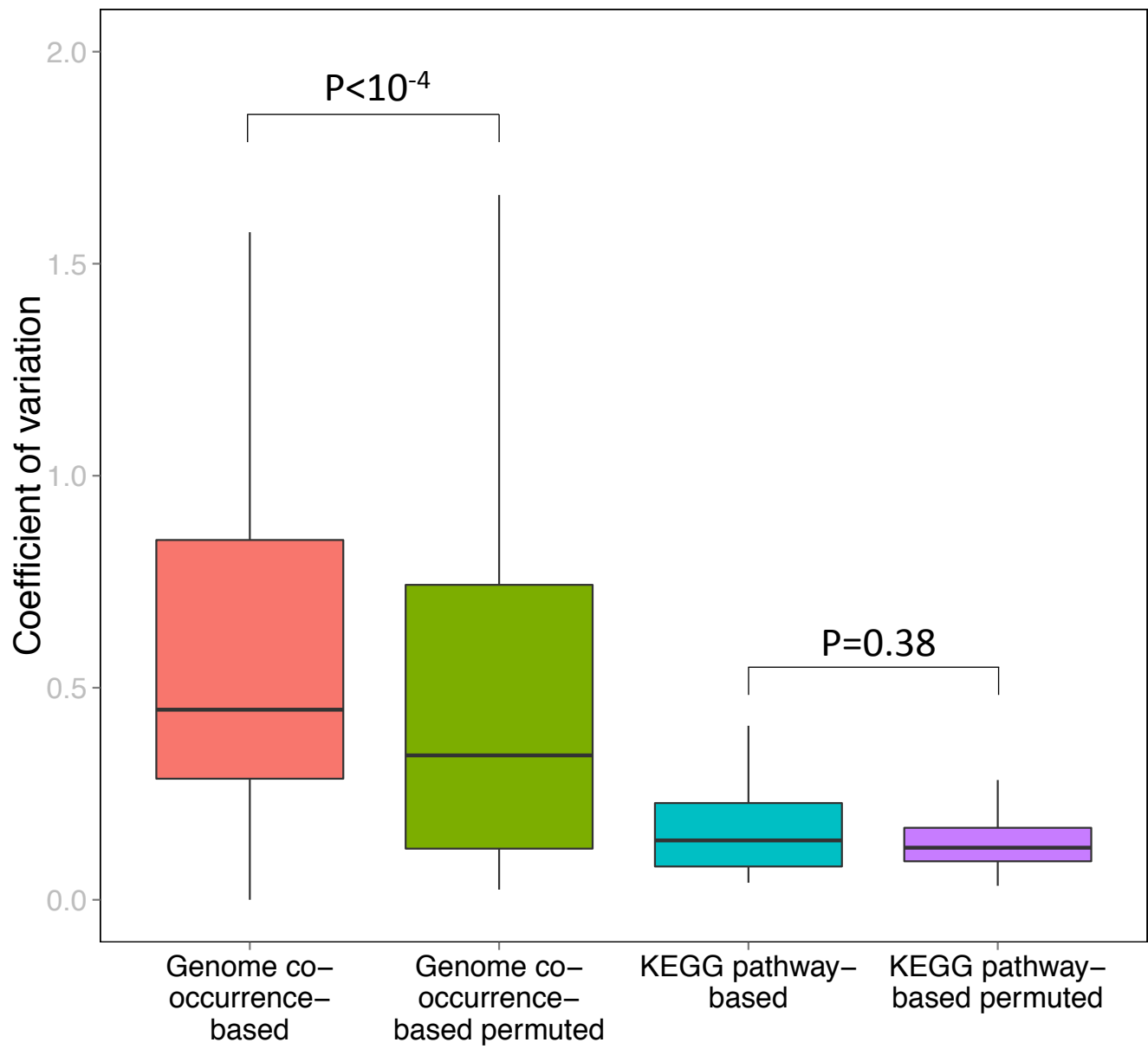

**Figure S5**

Supplement: Additional file 7: Figure S5. — Metagenomic functional variation in gene family aggregates based on KEGG pathways or microbial genome co-occurrence. Shown are boxplots of the distributions of Coefficient of Variation (CoV) values in the abundance of genome co-occurrence-based gene family aggregates and KEGG pathway-based gene family aggregates across gut samples from HMP. Since the average size of aggregates (i.e., the number of gene families assigned to each aggregate) is different between the two aggregation schemes, a permuted assignment of gene families to aggregates was performed for each scheme (i.e., preserving the size of the aggregates) and the CoV was calculated for the permuted aggregates. Notably, KEGG pathway-based aggregates did not show significantly higher values of CoV than KEGG pathway-based permuted aggregates (P=0.38, Wilcoxon rank-sum test), while genome co-occurrence-based aggregates showed significantly higher values of CoV than genome co-occurrence-based permuted aggregates (P<10-4, Wilcoxon rank-sum test). (PDF 251 kb) [file 40168_2017_231_MOESM7_ESM.pdf]

a

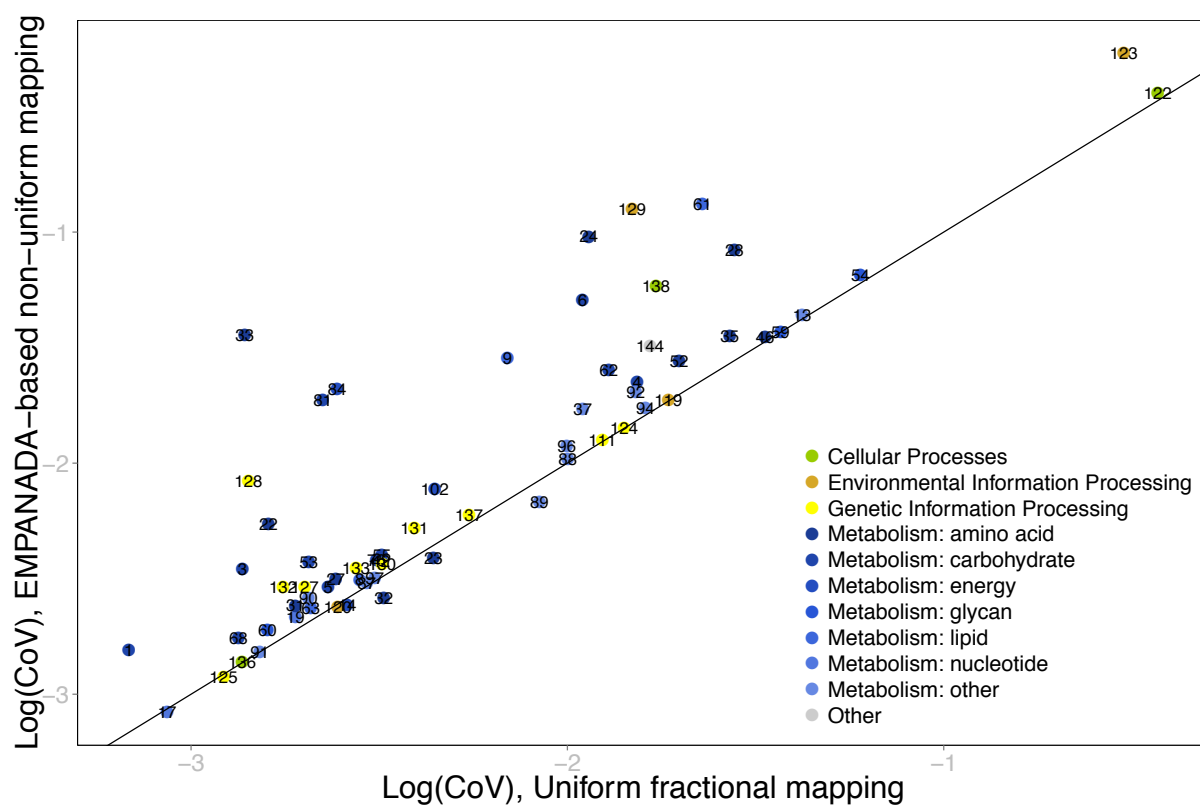

b

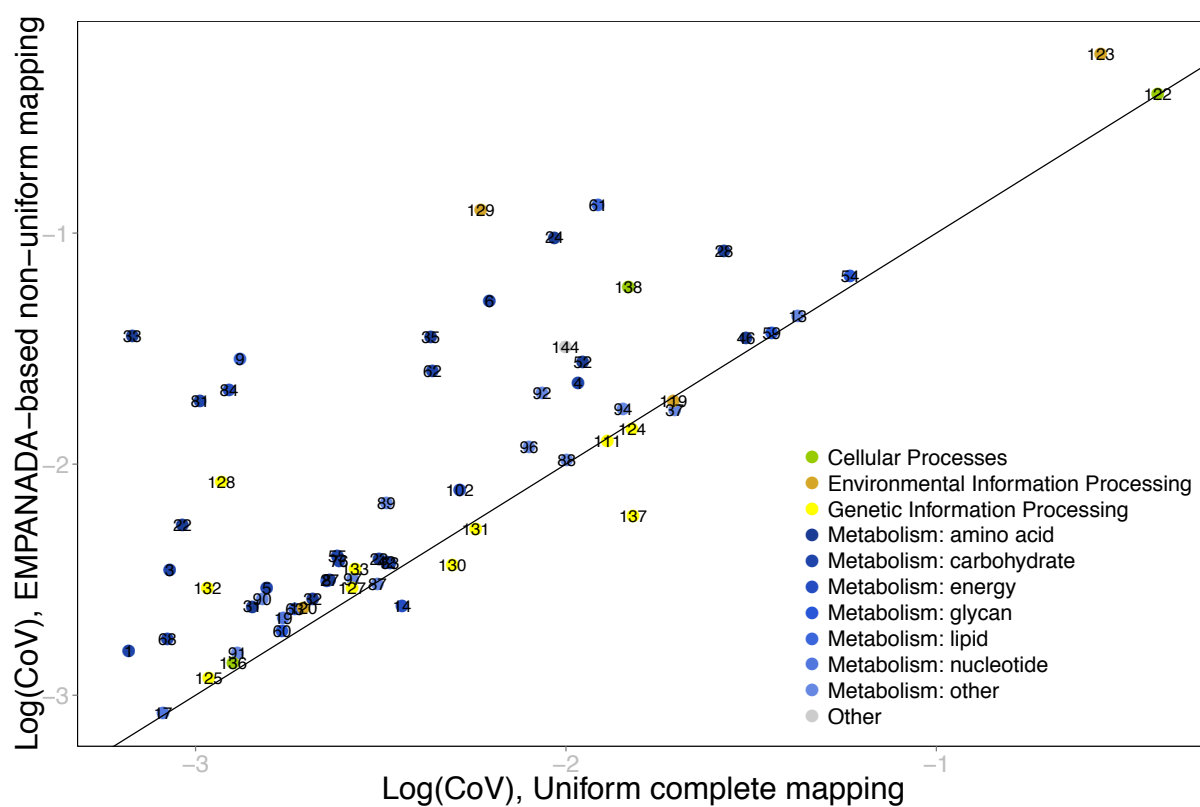

Figure S6

Supplement: Additional file 8: Figure S6. — Non-uniform and sample-specific mapping of gene families to pathways increases variability in the abundance of pathways in HMP gut samples. Shown is the same plot as in Fig. 4, with pathways additionally marked by a numeric identifier corresponding to their identifier in Additional file 3: Table S1, comparing the EMPANADA-based non-uniform mapping strategy with the uniform fractional (a) and uniform complete (b) mapping schemes. (PDF 251 kb) [file 40168_2017_231_MOESM8_ESM.pdf]

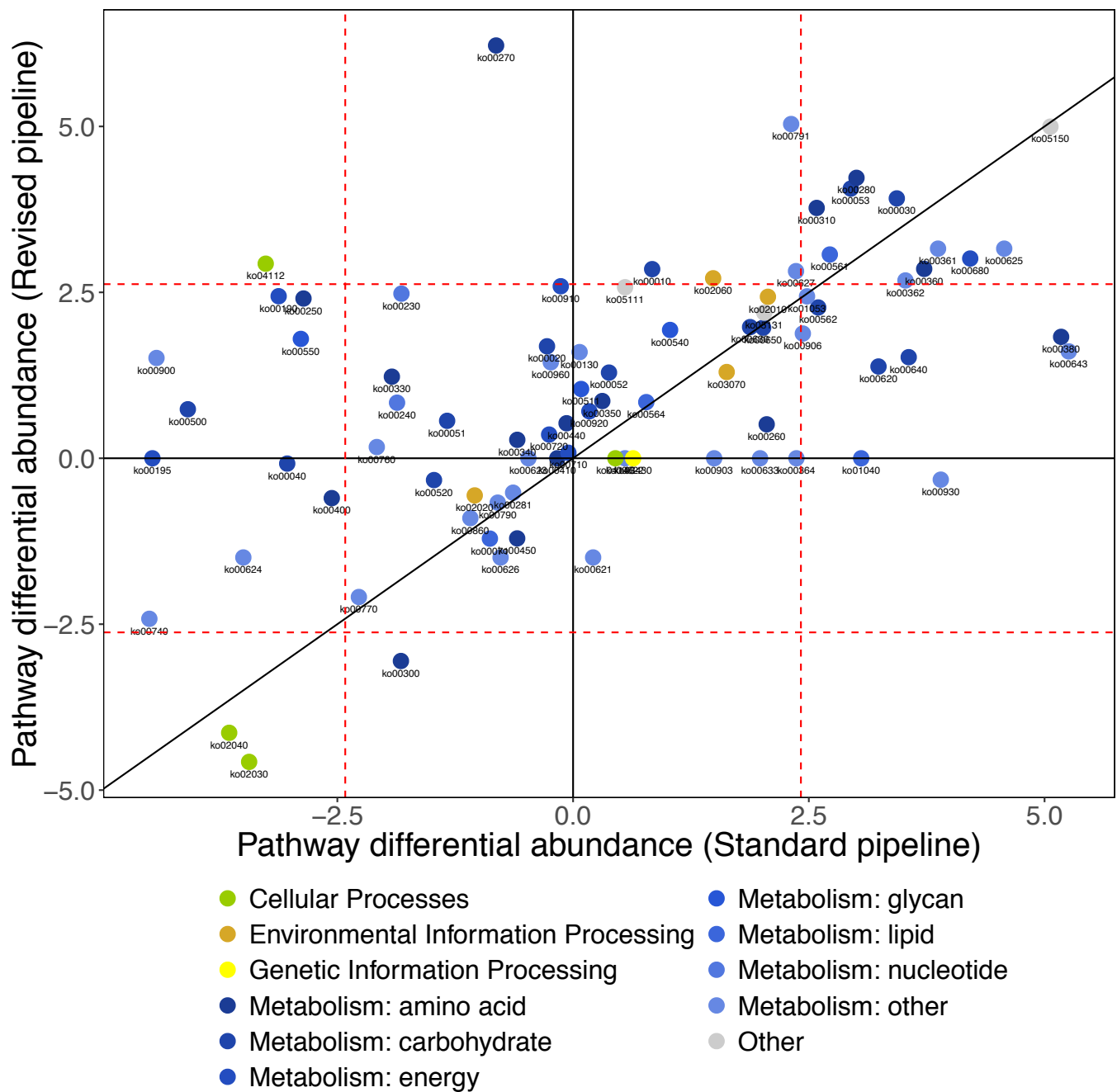

**Figure S7**

Supplement: Additional file 9: Figure S7. — Association of pathways to type 2 diabetes (T2D) with and without accounting for factors masking functional variability. Shown is a scatter plot of the differential abundance score (W-statistic of the Wilcoxon rank-sum test) of each pathway when comparing T2D cases and controls, using either the standard (x-axis) or revised (y-axis) metagenomic bioinformatic processing pipelines. Each point represents a single pathway and is additionally marked by its KEGG identifier. The dashed red lines represent the FDR<0.05 significance cutoff for each metagenomic processing pipeline. (PDF 251 kb) [file 40168_2017_231_MOESM9_ESM.pdf]

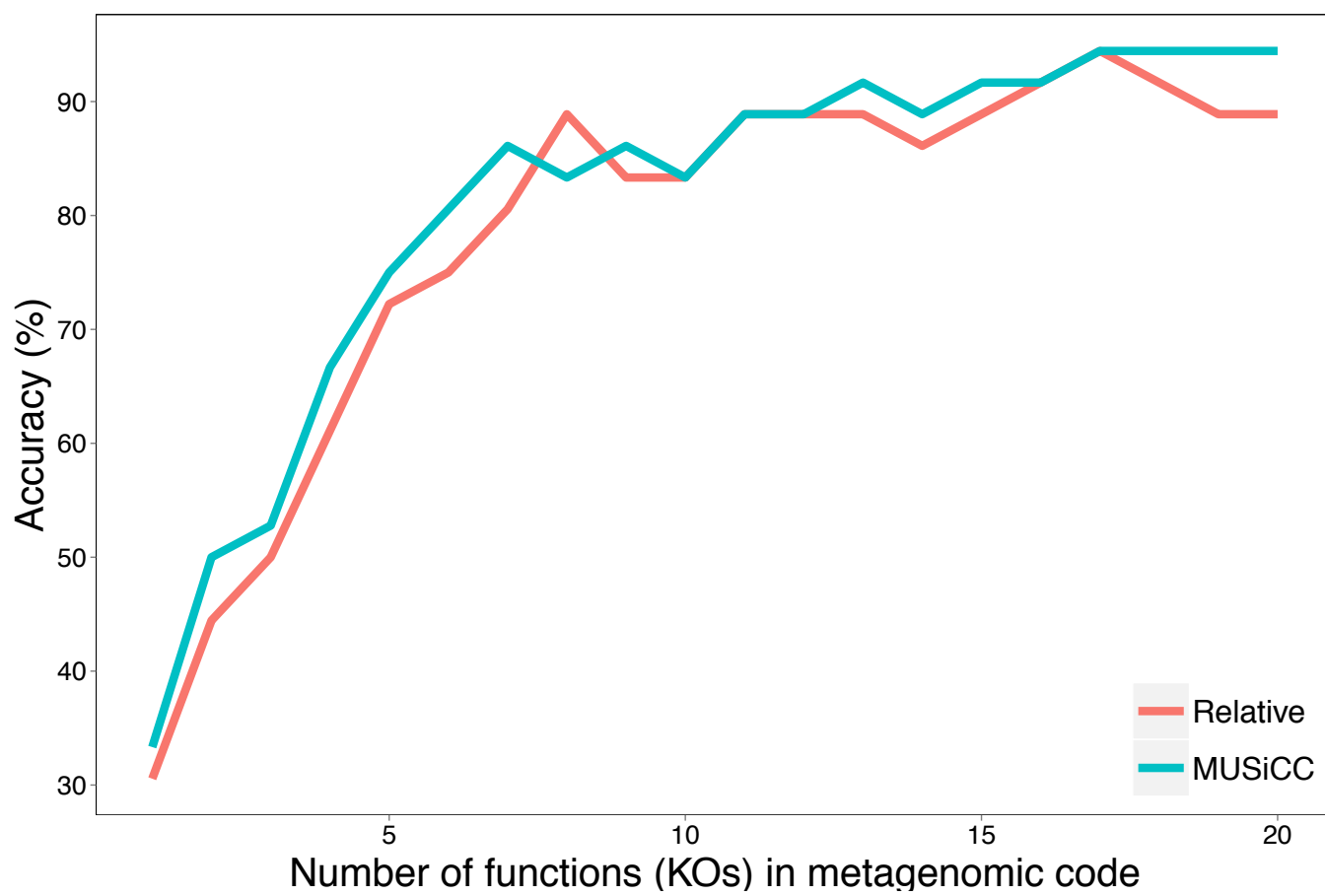

**Figure S8**

Supplement: Additional file 10: Figure S8. — Function-based metagenomic code. Shown is a plot of the accuracy of identification of an individual’s microbiome sample based on a previously obtained sample from the same individual. The identification was done using functional metagenomic codes of varying sizes (1-20 gene families), based on the gene family abundance profiles normalized with either relative (red) or MUSiCC (cyan) normalization procedures. (PDF 251 kb) [file 40168_2017_231_MOESM10_ESM.pdf]

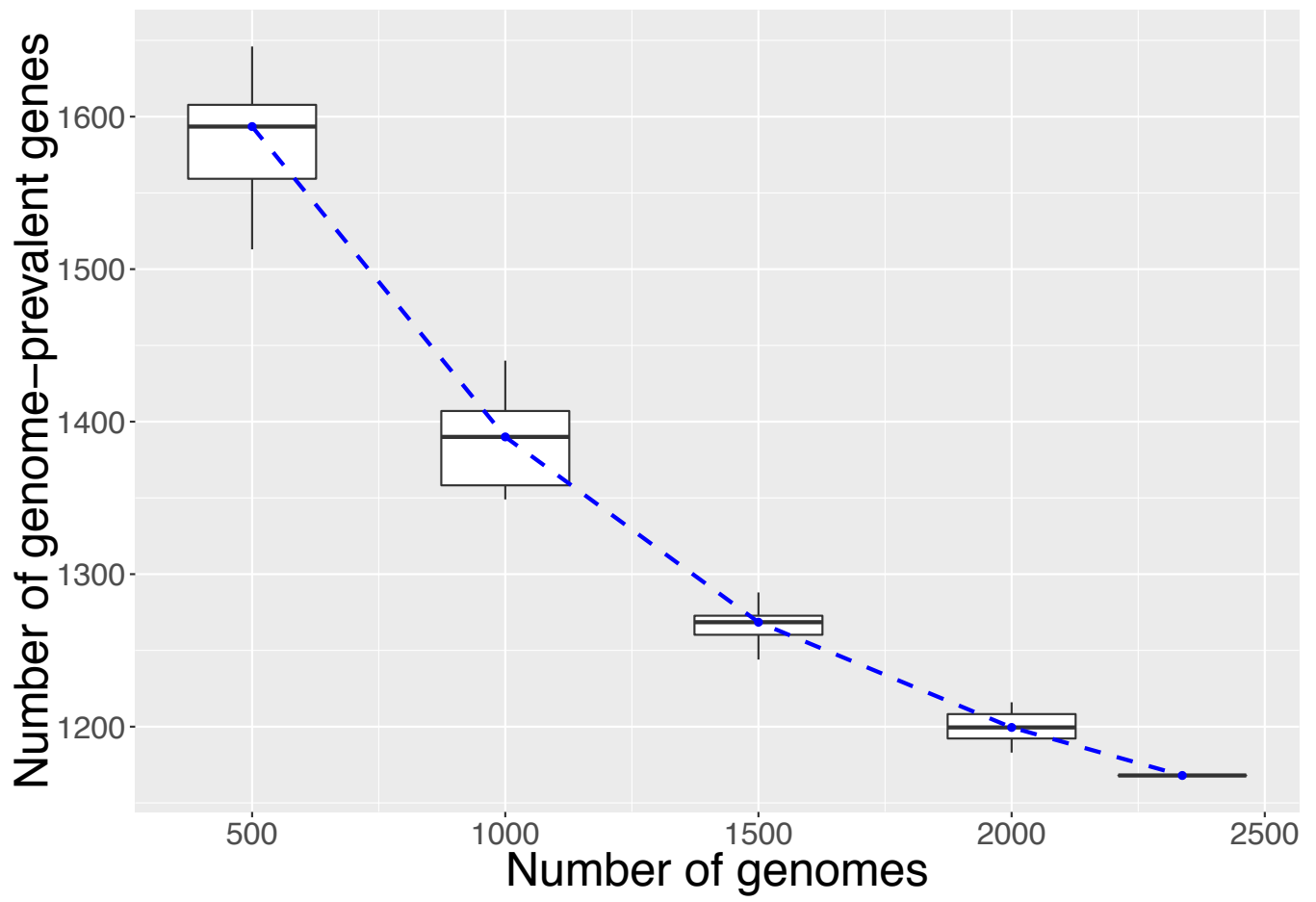

**Figure S9**

Supplement: Additional file 11: Figure S9. — Rarefication analysis of the number of all-genome prevalent genes as a function of the number of genomes used. All-genome prevalent genes were defined as KOs with a copy number CoV < 1.5 across bacterial genomes used. (PDF 251 kb) [file 40168_2017_231_MOESM11_ESM.pdf]
