# Supplementary material for: Revised computational metagenomic processing uncovers hidden and biologically meaningful functional variation in the human microbiome
Source: Microbiome. 2017 Feb 8;5:19. doi: 10.1186/s40168-017-0231-4 (PMC5299786; doi:10.1186/s40168-017-0231-4)
Supplement: Additional file 2: Figure S2. — Relative normalization masks functional variation of modules in HMP gut samples. Shown is a scatter plot similar to Fig. 1a but on the module level, where each module is additionally marked by a numeric identifier corresponding to its identifier in Additional file 3: Table S2. (PDF 251 kb) [file 40168_2017_231_MOESM2_ESM.pdf]

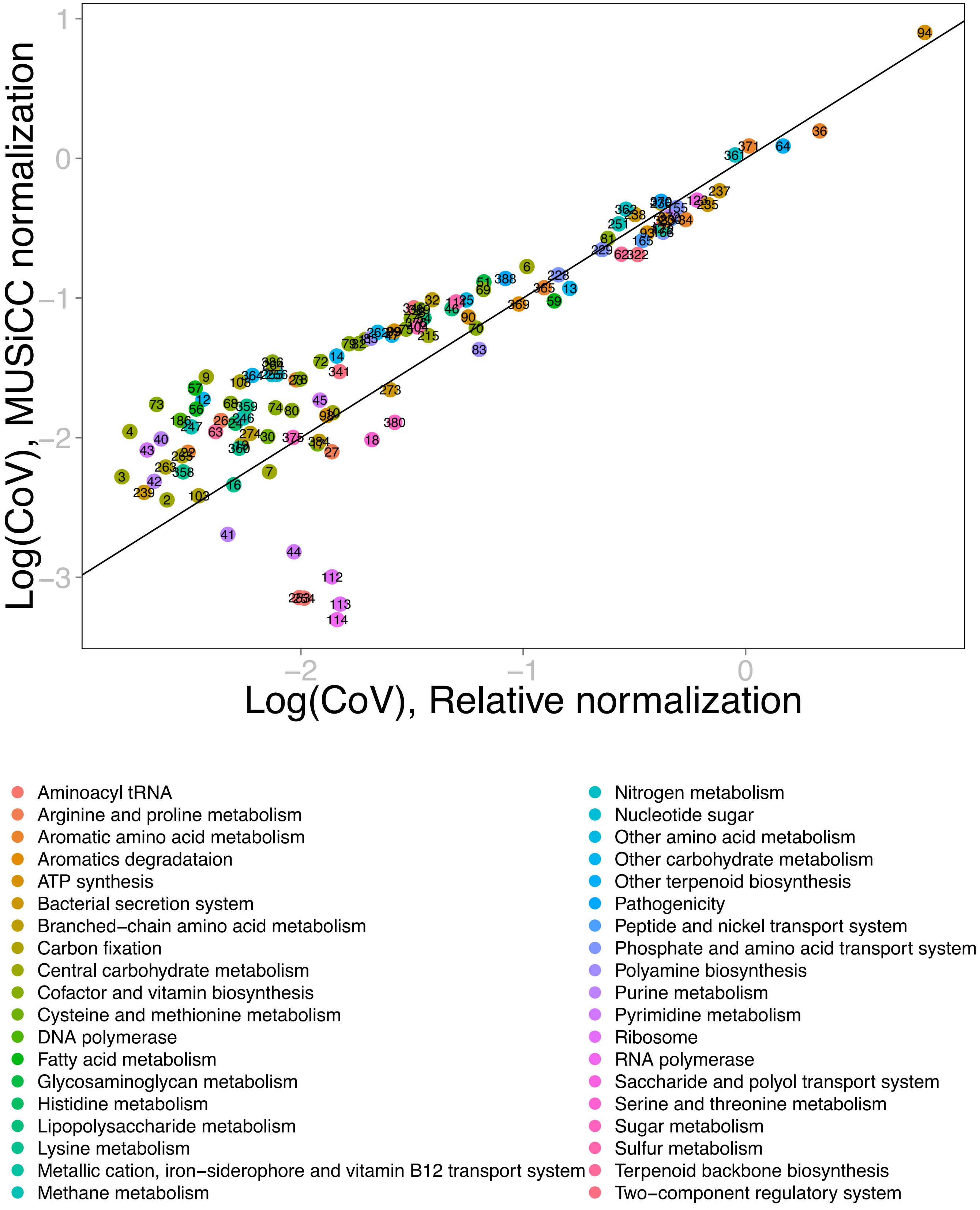
Log(CoV), MUSiCC normalization
Log(CoV), Relative normalization
1
0
−1
−2
−3
−2
−1
0
Aminoacyl tRNA
Arginine and proline metabolism
Aromatic amino acid metabolism
Aromatics degradataion
ATP synthesis
Bacterial secretion system
Branched−chain amino acid metabolism
Carbon fixation
Central carbohydrate metabolism
Cofactor and vitamin biosynthesis
Cysteine and methionine metabolism
DNA polymerase
Fatty acid metabolism
Glycosaminoglycan metabolism
Histidine metabolism
Lipopolysaccharide metabolism
Lysine metabolism
Metallic cation, iron−siderophore and vitamin B12 transport system
Methane metabolism
Nitrogen metabolism
Nucleotide sugar
Other amino acid metabolism
Other carbohydrate metabolism
Other terpenoid biosynthesis
Pathogenicity
Peptide and nickel transport system
Phosphate and amino acid transport system
Polyamine biosynthesis
Purine metabolism
Pyrimidine metabolism
Ribosome
RNA polymerase
Saccharide and polyol transport system
Serine and threonine metabolism
Sugar metabolism
Sulfur metabolism
Terpenoid backbone biosynthesis
Two−component regulatory system

Figure S2
