# Supplementary material for: Revised computational metagenomic processing uncovers hidden and biologically meaningful functional variation in the human microbiome
Source: Microbiome. 2017 Feb 8;5:19. doi: 10.1186/s40168-017-0231-4 (PMC5299786; doi:10.1186/s40168-017-0231-4)
Supplement: Additional file 4: Text S1. — Supplementary information for this study. (PDF 322 kb) [file 40168_2017_231_MOESM4_ESM.pdf]

Revised computational metagenomic processing uncovers hidden and biologically meaningful functional variation in the human microbiome

Ohad Manor and Elhanan Borenstein

Supplemental Information

MUSiCC normalization of simulated metagenomic samples unmasks true underlying variation

Using a large-scale simulation analysis, we set out to confirm that normalizing samples using MUSiCC can reveal true functional variation that is masked by relative normalization. To this end, we randomly selected a set of 10 reference genomes from KEGG, and used these genomes to construct 100 simulated samples (communities) with varying abundances for each reference genome in each sample. We then calculated the true absolute abundance of each functional pathway in each simulated sample, and computed the true Coefficient of Variation (CoV) for each pathway across all samples. Next, we normalized each sample using either relative normalization (*i.e.*, dividing the absolute abundance of each pathway by the total sum of pathway abundances) or MUSiCC, and re-calculated the CoV for each pathway across samples observed with each normalization method. This process was repeated 5 times with random selections of 10 reference genomes and simulation of 100 samples. Comparing the true CoV to the CoV observed in the normalized samples, we found that MUSiCC indeed recovered the true variation almost perfectly (median Spearman correlation between true and measured CoV across all pathways $\rho>0.99$), whereas relative normalization resulted in a significant bias in the observed CoV of functional pathways (median Spearman correlation $\rho=0.8$ across all pathways). We further repeated this analysis using communities with increased complexity, comprising of 50 or 100 reference genomes, and obtained a similar result (median Spearman correlation of $\rho>0.99$ for MUSiCC, *vs.* $\rho=0.9$, $\rho=0.92$ for relative normalization, for 50 and 100 reference genomes, respectively). Interestingly, we found that in these simulated communities, the pathways that were most affected by relative normalization (in terms of their CoV) were the *Ribosome* and *Glycolysis/Gluconeogenesis* pathways, which were also found to have markedly different CoV when using relative *vs.*

MUSiCC normalization in our analysis of HMP stool samples (see Supplementary Figure S3 and Figure 1a).

Standard and revised metagenomic processing pipelines identify different T2D functional associations

Applying the standard and revised metagenomic processing pipelines to a cohort of type 2 diabetes (T2D) cases and controls, we found that overall, the differential abundance patterns observed in the two pipelines are significantly correlated ($R=0.52$, $P<10^{-7}$, Pearson correlation test across all functional pathways). Notably, however, several pathways showed a different T2D-assocaition pattern in the two pipelines (Supplementary Figure S7), with some pathways identified as T2D-enriched only in the revised pipeline (see main text), and 6 pathways showing association with T2D only in the standard pipeline. These pathways include Biosynthesis of unsaturated fatty acids (ko01040), Caprolactam degradation (ko00930), Pyruvate metabolism (ko00620), Propionate metabolism (ko00640), Tryptophan metabolism (ko00380), and Styrene degradation (ko00643). Indeed, increased Tryptophan metabolism in the microbiome has been associated with T2D in a previous study [1], yet this same study identified Pyruvate metabolism as *depleted* in T2D (*i.e.*, associated with control samples), and Propionate metabolism to be a spurious T2D enrichment that was derived from not accounting for treatment with metformin. Studies that link Styrene degradation, Caprolactam degradation, and Biosynthesis of unsaturated fatty acids in the microbiome to T2D are also lacking. Without a clear gold standard for pathways associated with T2D in the microbiome, it is of course challenging to determine if the associations identified above by the standard pipeline and not by the revised pipeline are true. Such associations may, for example, be missed by the revised pipeline due to reduced power accompanying the filtration of prevalent genes, and additional studies focusing on the mechanism linking microbial pathways to T2D etiology are needed to confidently determine such associations.

Supplemental References

1. Forslund K, Hildebrand F, Nielsen T, Falony G, Le Chatelier E, Sunagawa S, et al. Disentangling type 2 diabetes and metformin treatment signatures in the human gut microbiota. Nature. Nature Publishing Group; 2015;528:262–6.
